# Densitometric and local histogram based analysis of computed tomography images in patients with idiopathic pulmonary fibrosis

**DOI:** 10.1186/s12931-017-0527-8

**Published:** 2017-03-07

**Authors:** Samuel Y. Ash, Rola Harmouche, Diego Lassala Lopez Vallejo, Julian A. Villalba, Kris Ostridge, River Gunville, Carolyn E. Come, Jorge Onieva Onieva, James C. Ross, Gary M. Hunninghake, Souheil Y. El-Chemaly, Tracy J. Doyle, Pietro Nardelli, Gonzalo V. Sanchez-Ferrero, Hilary J. Goldberg, Ivan O. Rosas, Raul San Jose Estepar, George R. Washko

**Affiliations:** 1 Division of Pulmonary and Critical Care Medicine, Department of Medicine, Brigham and Women’s Hospital, 75 Francis St., PBB, CA-3, Boston, MA 02115 USA; 2 Laboratory of Mathematics in Imaging, Department of Radiology, Brigham and Women’s Hospital, 1249 Boylston St, Boston, MA 02115 USA; 3 NIHR Southampton Respiratory Biomedical Research Unit, Southampton Centre for Biomedical Research, Southampton General Hospital, Tremona Road MP218, Southampton, SO16 6YD UK; 4 Department of Biology, Creighton University, 2500 California Plaza, Omaha, NE 68178-0324 USA

**Keywords:** Interstitial lung disease, Idiopathic pulmonary fibrosis, Computed tomography, Quantitative, Imaging, Mortality

## Abstract

**Background:**

Prior studies of clinical prognostication in idiopathic pulmonary fibrosis (IPF) using computed tomography (CT) have often used subjective analyses or have evaluated quantitative measures in isolation. This study examined associations between both densitometric and local histogram based quantitative CT measurements with pulmonary function test (PFT) parameters and mortality. In addition, this study sought to compare risk prediction scores that incorporate quantitative CT measures with previously described systems.

**Methods:**

Forty six patients with biopsy proven IPF were identified from a registry of patients with interstitial lung disease at Brigham and Women’s Hospital in Boston, MA. CT scans for each subject were visually scored using a previously published method. After a semi-automated method was used to segment the lungs from the surrounding tissue, densitometric measurements including the percent high attenuating area, mean lung density, skewness and kurtosis were made for the entirety of each patient’s lungs. A separate, automated tool was used to detect and quantify the percent of lung occupied by interstitial lung features. These analyses were used to create clinical and quantitative CT based risk prediction scores, and the performance of these was compared to the performance of clinical and visual analysis based methods.

**Results:**

All of the densitometric measures were correlated with forced vital capacity and diffusing capacity, as were the total amount of interstitial change and the percentage of interstitial change that was honeycombing measured using the local histogram method. Higher percent high attenuating area, higher mean lung density, lower skewness, lower kurtosis and a higher percentage of honeycombing were associated with worse transplant free survival. The quantitative CT based risk prediction scores performed similarly to the clinical and visual analysis based methods.

**Conclusions:**

Both densitometric and feature based quantitative CT measures correlate with pulmonary function test measures and are associated with transplant free survival. These objective measures may be useful for identifying high risk patients and monitoring disease progression. Further work will be needed to validate these measures and the quantitative imaging based risk prediction scores in other cohorts.

**Electronic supplementary material:**

The online version of this article (doi:10.1186/s12931-017-0527-8) contains supplementary material, which is available to authorized users.

## Background

Idiopathic pulmonary fibrosis (IPF) is a progressive and usually fatal disease. While the median survival is only 2.53.5 years without treatment, the clinical course can be variable, and the prediction of IPF related outcomes in an individual can be challenging [1, 2]. IPF severity is often described using pulmonary function test (PFT) parameters, or using systems such as the GAP index, which incorporates gender, age, forced vital capacity (FVC) and diffusing capacity (DLCO) into a risk prediction model [3]. Because patients with IPF usually have had a computed tomography (CT) scan, both qualitative and quantitative CT measurements have also been studied to determine their associations with PFT measures and clinical outcomes [4–15]. These studies have often used volumetric CT data acquired in a research setting, and there has been limited investigation into what additional information CT measurements provide above and beyond risk prediction models such as the GAP index [16].

In this study, using clinically acquired non-volumetric data, we aimed to validate previously described associations of densitometric quantitative CT measurements with PFT parameters and mortality in IPF. [6, 17] In addition, we have developed a fully automated method that uses the local histogram pattern of lung density combined with the distance from the pleural surface to quantify the volume of radiographic tissue subtypes that make up normal, emphysematous, and interstitial tissues [18, 19]. We hypothesized that the total percentage of interstitial disease and the percentage of disease that was the honeycombing subtype would be associated with FVC, DLCO and mortality in IPF. In addition, we aimed to compare the prognostic value of a previously described clinical and visual analysis based risk prediction system with those based on clinical and objective measures [16]. Specifically, because a visually calculated fibrosis score had been previously described to be able to substitute for diffusing capacity in the GAP score, we hypothesized that the objectively measured interstitial changes, measured either directly using a local histogram based detection system, or indirectly in the form of high attenuation areas, could be used in a similar manner [4, 16, 20]. Finally, because honeycombing has been shown to be related to poor outcomes in IPF, we hypothesized that the percentage of interstitial disease that was honeycombing could be used in the same way [4].

## Methods

### Study design and data acquisition

The David E. Herlihy Data Registry and DNA Repository is an institutional review board approved registry of patients with ILD at Brigham and Women’s Hospital in Boston, MA (Partners Institutional Review Board Protocol Number 2012-P-000840/1). All patients over the age of 18 receiving care for interstitial lung disease (ILD) at Brigham and Women’s Hospital are eligible to enroll. At the time of enrollment patients have the option to opt in or out of the 5 portions of the study including a research questionnaire, medical history review, serum and plasma banking, genetic testing, and future contact for other studies. All patients in the registry provided informed consent and only those patients who consented to medical history review, which specifically includes the analysis of imaging data, were considered for this study. Only those patients who had a prior lung biopsy that showed usual interstitial pneumonia (UIP), and who had CT imaging and spirometry within 48 h of each other were included. Patients whose longitudinal follow up data were not available, including lung transplant status and mortality, were excluded. All testing was performed only as clinically indicated.

### Objective CT analyses

The CT images used for this study were those typically termed “high resolution”: non-volumetric 1 mm slices with 10 mm spacing with a sharp kernel image reconstruction obtained without the administration of intravenous contrast. Using a previously described automated technique, the lung was segmented from the surrounding tissue [21]. The axial images were then visually inspected and manually edited as needed to correct inaccurate segmentations.

**Fig. 1:**
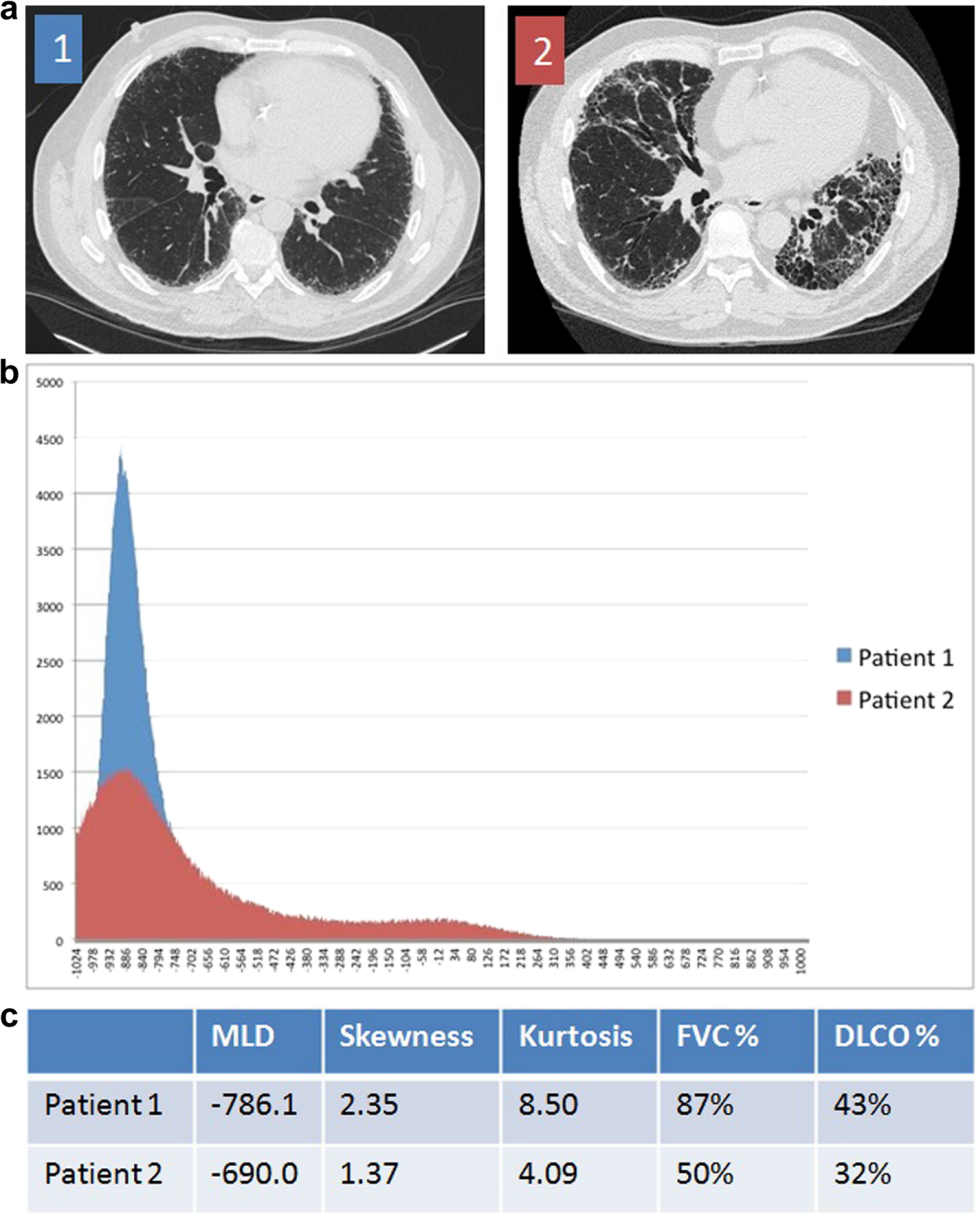
**a** Representative images from subjects with less severe (Patient 1) and more severe (Patient 2) visual evidence of IPF. **b** Histograms of distribution of the number of voxels on the y axis for each tissue density in Hounsfield Units on the x axis. **c** Summary statistics and selected pulmonary function test parameters for each subject. Abbreviations: mean lung density (MLD)

For the densitometric evaluation, the histogram of distribution of the density of each voxel within the lung was plotted as shown in Fig. 1, and the skewness, kurtosis, and the mean of that distribution (mean lung density, MLD) were measured. In addition, the percentage of the total volume of tissue that had a density between -250 Hounsfield units (HU) and -600 HU was recorded as the percent high attenuation area (HAA%) [22].

**Fig. 2:**
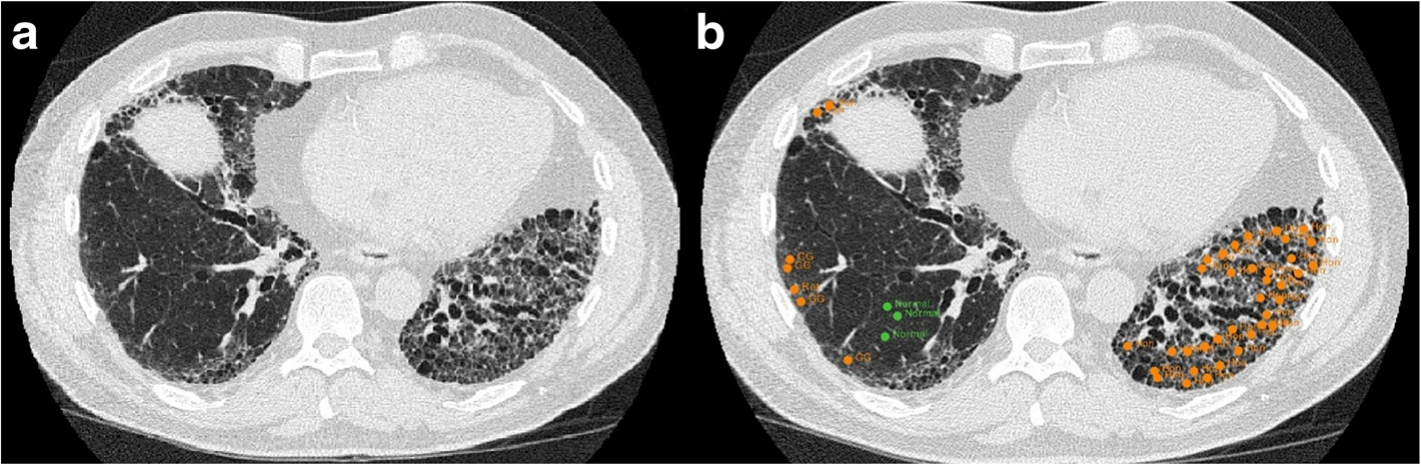
**a** Sample slice for CT scan of a subject. **b** The same sample slice from a subject CT scan showing placement of fiducials for the training of the local histogram based objective method. Abbreviations: ground glass (GG), honeycombing (Hon), reticular (Ret), computed tomography (CT)

**Fig. 3:**
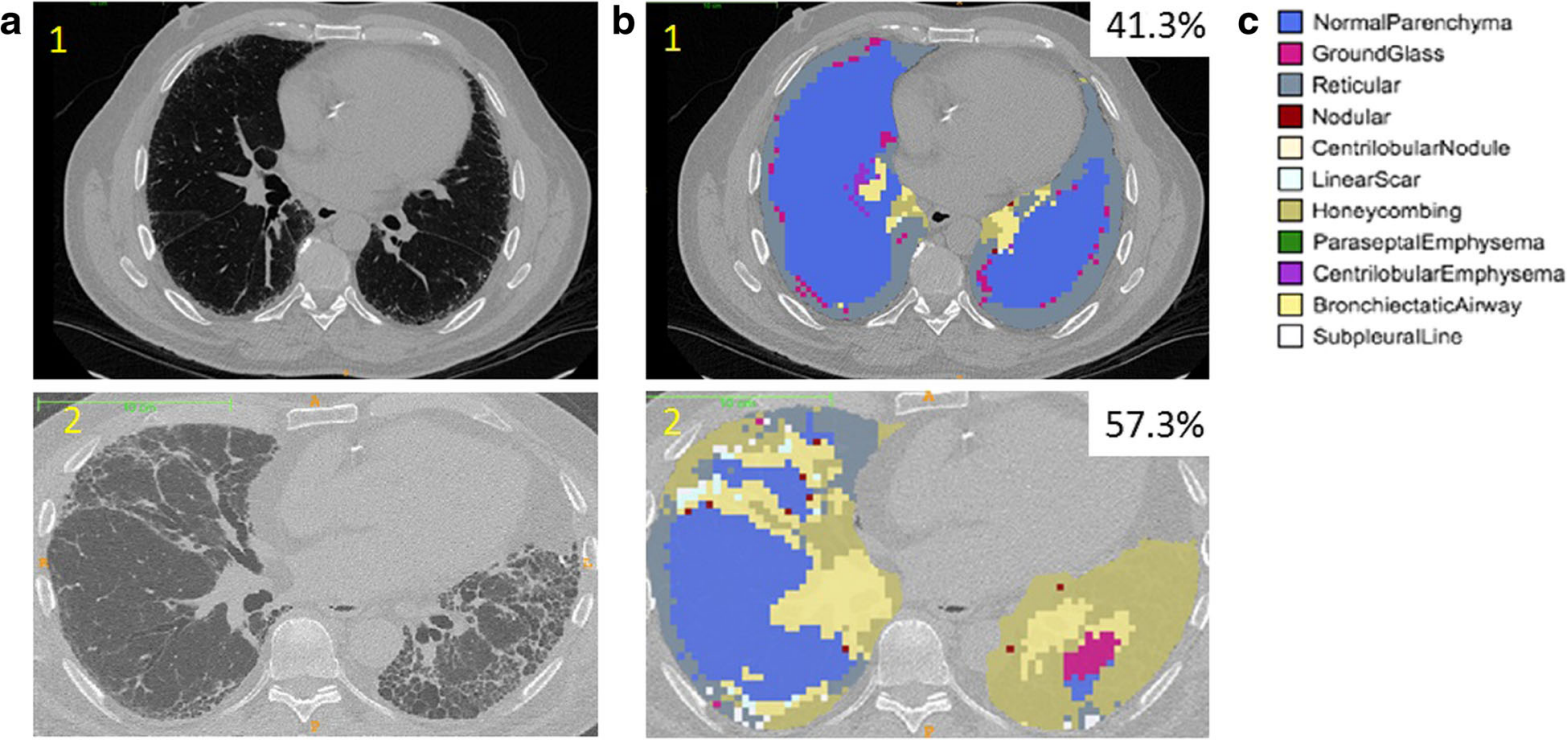
**a** Representative CT images from subjects with less severe IPF (patient 1) and more severe IPF (patient 2). **b** Overlay of categorization of lung parenchyma into radiographic subtypes using the local histogram analysis and distance based analysis for each subject. **c** Legend for radiographic subtypes

Full details regarding the local histogram based objective quantification of the volume of radiographic feature subtypes are available in the (Additional file 1). Briefly, we used both the properties of the local tissue and the distance from the pleural surface to determine a radiographic feature subtype for every portion of the lung [18, 19, 23]. First, in order to train the subtype identification tool, a single expert placed a total of 3357 fiducials, in 30 randomly selected subjects, on the following radiographic subtypes: normal, interstitial (reticular, centrilobular nodule, linear scar, nodular, subpleural line, ground glass and honeycombing), and emphysematous (centrilobular and panlobular) as shown in Fig. 2.^1^ This was done to build a library of points to be used as tissue classifiers. Regions of interest consisting of 30 by 30 in-plane voxels were built around these training points, and both the local histogram information and distance from the pleural surface were used to create a tissue classification vector for each region [18, 19]. After the training process was completed, the feature vectors of all of the 30 by 30 in-plane voxel regions within the lungs of each of the subjects were classified into tissue subtypes based on their similarity to the training data as shown in Fig. 3.

**Fig. 4:**
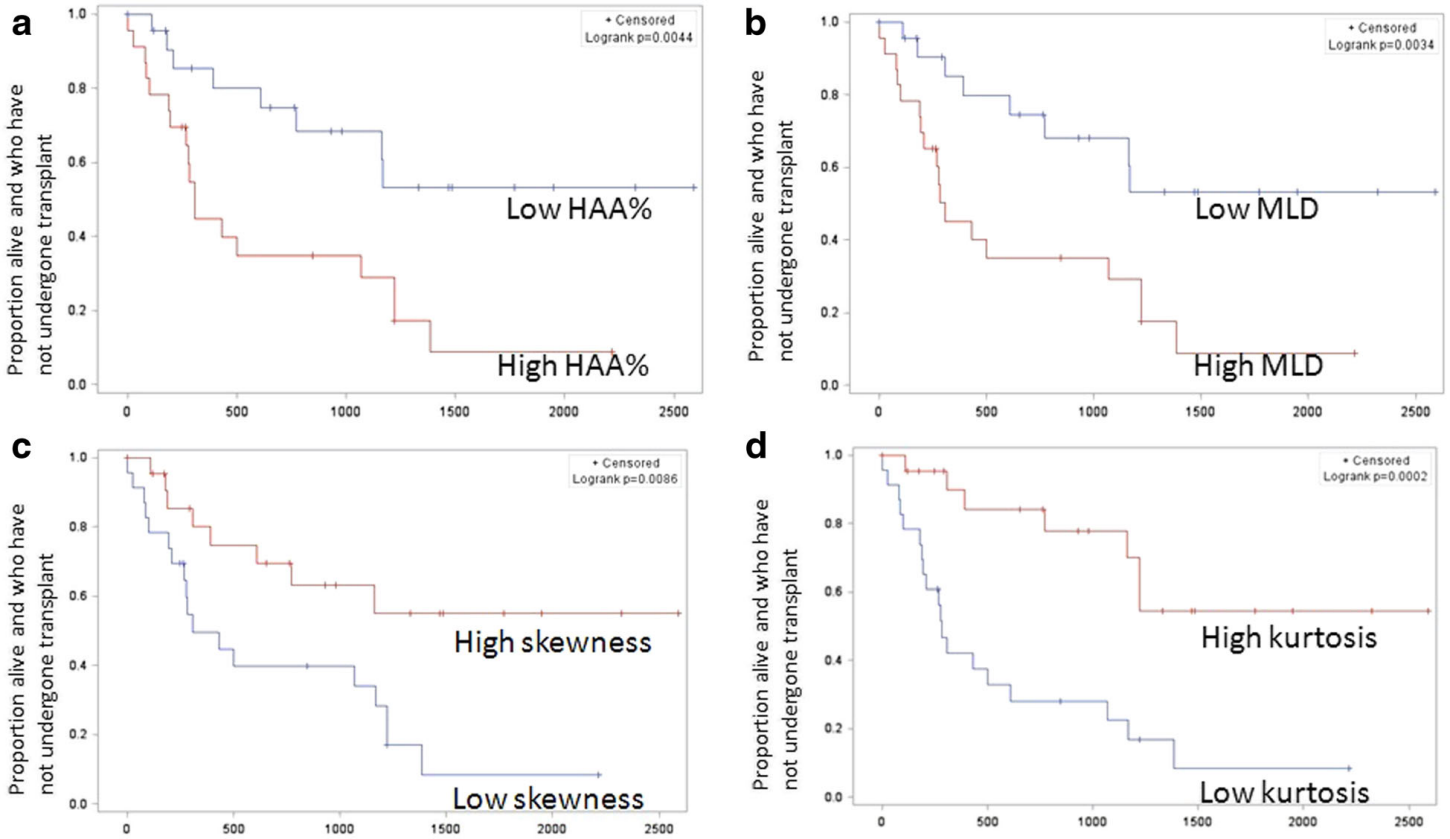
Kaplan Meier survival curves for transplant free survival for the densitometric CT measures. **a** HAA%, **b** MLD, **c** skewness, **d** kurtosis. Abbreviations: percentage of high attenuation area (HAA%), mean lung density (MLD)

The total percentage of all of the interstitial features (objective interstitial score, interstitial%) was then determined by combining the reticular, centrilobular nodule, linear scar, nodular, subpleural line, ground glass and honeycombing subtype volumes and dividing by the total volume of all tissue types (normal, interstitial and emphysematous). The percentage of interstitial disease made up of by honeycombing (honeycombing%) was determined by dividing the volume of the honeycombing subtype by the total volume of all of the interstitial subtypes. Due to the exploratory nature and small size of this study, subjects used in the training set were not excluded from the final analysis.

### Visual analysis

The same CT images used for the objective analysis were reviewed by two experts and the percentage of lung occupied by fibrotic disease (reticular and honeycombing) was qualitatively defined as described previously [16, 24]. Briefly, the two experts determined the percentage of lung occupied by reticular and honeycombing changes in each of six lung zones, three on each side, with the upper lung zone extending from the apex to the level of the aortic arch, the middle from the aortic arch to the inferior pulmonary veins, and the inferior from the inferior pulmonary veins to the bases. The percentages identified by both experts were then averaged to determine the mean total percentage of lung occupied by visually defined fibrotic changes, also termed the visual fibrosis score (fibrosis%) [16, 24].

### Statistical analysis

Pearson correlation coefficients were calculated between the CT measurements (densitometric: MLD, skewness, kurtosis, HAA%, visual: fibrosis%, and local histogram based: interstitial%, honeycombing%) and the nearest in time percent predicted forced vital capacity (FVC%) and percent predicted diffusing capacity of carbon monoxide (DLCO%). The Pearson correlation coefficient was also calculated between the visual fibrosis score (fibrosis%) and the objective interstitial score (interstitial%).

All of the CT predictors were dichotomized at their medians and both Kaplan-Meier and univariate Cox regression survival analyses were performed for the outcome of time to death or transplant (transplant free survival). Univariate Cox regression survival analyses were also performed for the outcome of time to death for the subgroup of subjects who did not undergo transplant. Each predictor was evaluated using the cumulative sum of Martingale-based residuals and none were found to violate the proportional hazards assumption [25].

The continuous GAP score was calculated for each subject as previously published [3]. For the purposes of the GAP score calculation, subjects who did not have a DLCO available were assumed to have been unable to perform the test. The continuous qualitative or visual GAP-CT score was calculated by using the visual fibrosis score in place of the DLCO as previously described [16]. Three additional GAP scores were calculated by using the following quantitative measures in place of the visual fibrosis score: 1) interstitial% (GAP-QCT), 2) honeycombing% (GAP-honeycombing), and 3) HAA% (GAP-HAA). For each of these combination clinical and imaging scoring methods, their performance with regard to the prediction of transplant free survival was assessed using the c-index, which was calculated using the method described by Pencina et al. [26, 27] The c-index accommodates censoring, and can be thought of as similar to the area under the receiver operating curve for logistic regression, in that it ranges from 0 to 1.0 (variably expressed as 0-100%), and a value of 0.5 indicates no predictive discrimination while a value of 1.0 indicates perfect discrimination [16].

A p value of less than 0.05 was considered to indicate statistical significance. All analyses were performed using SAS, version 9.4 (SAS Institute, Cary, NC).

## Results

Of the 176 subjects in the registry as of August 15, 2015, 46 (26%) met inclusion criteria. All 46 had longitudinal data available regarding lung transplant and mortality, and 36 (78%) had DLCO measurements available for the same time point as spirometry. The clinical characteristics of these subjects are shown in Table 1. While the indications for lung biopsy were not recorded in all cases, visual review revealed that the majority of subjects had CT findings consistent with UIP.

**Table 1:** Characteristics of the cohort

Gender	Number	Percent
Male	33	71.7
Race	Number	Percent
White	38	82.6
Black	2	4.35
Asian	3	6.5
Hispanic	3	6.5
Pulmonary Function	Mean (Standard Deviation)	Median, Interquartile Range
FEV1 - L	2.03 (0.69)	2.02, [1.62, 2.45]
FEV1 % predicted	64.76 (21.05)	64, [49, 82]
FVC - L	2.40 (0.86)	2.36, [1.71, 2.91]
FVC % predicted	60.17 (21.11)	58.5, [44.0, 73.0]
DLCO – mL/min/mmHg	10.40 (3.99)	10.06, [7.75, 12.53]
DLCO % predicted	39.61 (15.66)	37.0, [30.5, 48.5]
Quantitative CT Measures - Densitometric	Mean (Standard Deviation)	Median, Interquartile Range
Mean Lung Density (HU)	-670.14 (86.24)	-680.46, [-733.31, -607.03]
Skewness	1.35 (0.55)	1.40, [1.01, 1.79]
Kurtosis	5.06 (2.32)	4.72, [3.18, 6.18]
Percentage of Lung Occupied by High Attenuation Area, HAA%	17.21 (6.45)	15.41, [12.54, 23.13]
Qualitative CT Measures	Mean (Standard Deviation)	Median, Interquartile Range
Fibrosis Score, fibrosis%	26.89 (15.80)	22.92, [14.17, 37.50]
Quantitative CT Measures - Feature Based	Mean (Standard Deviation)	Median, Interquartile Range
Quantitative interstitial score, interstitial%	68.53 (18.39)	65.75, [57.53, 81.90]
Percentage of Interstitial Disease Occupied by Honeycombing, Honeycombing%	26.36 (17.19)	21.65, [12.09, 38.04]

*Abbreviations*: computed tomography (*CT*), diffusing capacity of carbon monoxide (*DLCO*), forced expiratory volume in 1 s (*FEV1*), forced vital capacity (*FVC*), Hounsfield units (*HU*), standard deviation (*SD*)

**Fig. 5:**
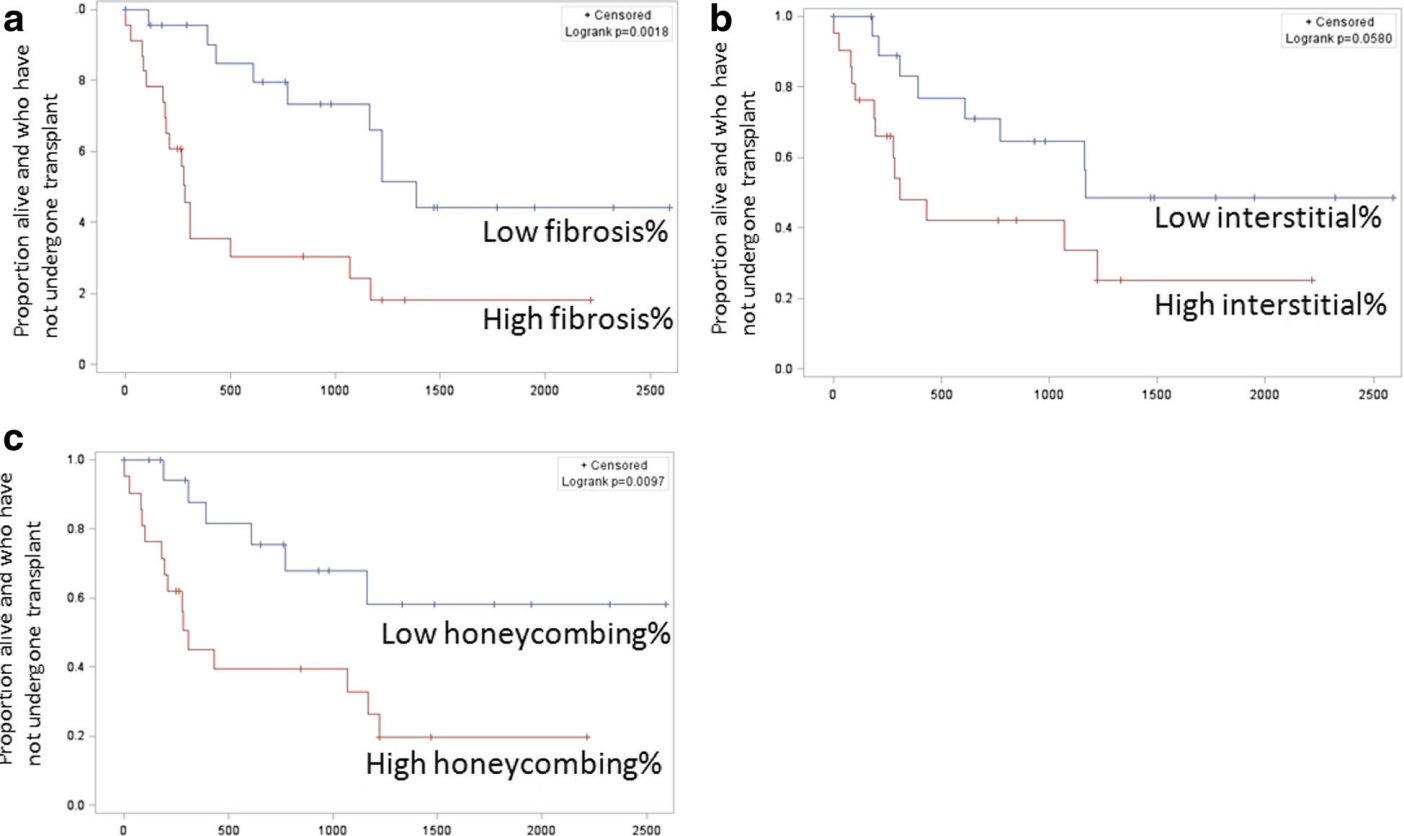
Kaplan Meier survival curves for transplant free survival for the visual fibrosis score (**a**), the objective interstitial score (**b**), and the percentage of interstitial disease that was honeycombing (**c**)

The visual fibrosis score and the objective interstitial score were highly correlated (r = 0.77, *p* < 0.001). All of the CT measurements, including the densitometric measures, the quantitative interstitial score, the quantitative honeycombing measure, and the visual fibrosis score were strongly correlated with both FVC% and DLCO% as shown in Table 2. The median follow up duration was 465 days, during which 14 (30%) patients underwent lung transplant and 12 (26%) died. Higher HAA%, higher MLD, lower skewness and lower kurtosis were associated with a shorter transplant free survival as shown in Fig. 4 a-d and Table 3. Those subjects with higher fibrosis% and honeycombing% also had a shorter transplant free survival as shown in Figs. 5a and c and Table 3. There was trend toward a shorter transplant free survival for those subjects with higher interstitial%, but this did not reach statistical significance as shown in Fig. 5b and Table 3. In the subgroup that did not undergo transplant, the same features were associated with increased mortality with the exception of honeycombing% as shown in Table 3.

**Table 2:** Pearson correlation coefficients for quantitative CT measures and PFT measures

CT measure	FVC % predicted		DLCO % predicted	
	r	p	r	p
Quantitative Densitometric				
Mean Lung Attenuation	-0.78	<0.001	-0.73	<0.001
Skewness	0.76	<0.001	0.73	<0.001
Kurtosis	0.71	<0.001	0.68	<0.001
Percent High Attenuating Area	-0.77	<0.001	-0.69	<0.001
Qualitative				
Fibrosis Score, fibrosis%	-0.64	<0.001	-0.7	<0.001
Quantitative Feature Based				
Quantitative Interstitial Score, interstitial%	-0.79	<0.001	-0.70	<0.001
Percentage of Interstitial Disease Occupied by Honeycombing, Honeycombing%	-0.66	<0.001	-0.66	<0.001

*Abbreviations*: computed tomography (*CT*), diffusing capacity of carbon monoxide (*DLCO*), forced vital capacity (*FVC*)

**Table 3:** Unadjusted hazard ratios for death or transplant (A) and for death in those who did not undergo transplant (B) for measurements dichotomized at their medians

Measurement	Hazard ratio for death or transplant	p	Hazard ratio for death^a^	p
Quantitative Densitometric				
Mean Lung Density (High vs. Low)	3.27	0.006	5.05	0.015
Skewness (Low vs. High)	2.92	0.012	3.56	0.039
Kurtosis (Low vs. High)	4.49	0.001	4.37	0.013
High Attenuating Area (High vs. Low)	3.17	0.007	7.69	0.009
Qualitative				
Fibrosis Score, fibrosis% (High vs. Low)	3.48	0.003	3.96	0.026
Quantitative Feature Based				
Quantitative Interstitial Score, interstitial% (High vs. Low)	2.30	0.065	3.51	0.065
Percentage of Interstitial Disease Occupied by Honeycombing, Honeycombing% (High vs. Low)	3.28	0.014	2.86	0.094
Pulmonary Function Test				
FVC percent predicted (Low vs. High)	2.80	0.016	3.14	0.063
DLCO percent predicted (Low vs. High)	3.78	0.012	3.38	0.100

*Abbreviations*: diffusing capacity of carbon monoxide (*DLCO*), forced vital capacity (*FVC*). ^a^In the subgroup who did not undergo transplantation

The c-indices for the standard GAP score as well as for the GAP-CT, GAP-QCT, GAP-honeycombing and GAP-HAA scores are shown in Table 4. With the exception of the GAP-HAA score all of the calculated continuous GAP scores had similar c-indices. These were also similar to the previously published c-indices for both the standard GAP score and the GAP-CT score [3, 20].

**Table 4:** c-Indices for continuous GAP models for prediction of transplant free survival

Predictor	c-Index	Confidence interval
Diffusing Capacity, DLCO	0.66	0.56, 0.75
High Attenuation Area, HAA%	0.59	0.50, 0.68
Visual Fibrosis Score, fibrosis%	0.69	0.61, 0.77
Quantitative Interstitial Score, interstitial%	0.65	0.54, 0.75
Percentage of Interstitial Disease Occupied by Honeycombing, Honeycombing%	0.62	0.52, 0.71

## Discussion

In this study of 46 subjects with biopsy proven IPF we found that both densitometric and local histogram based quantitative CT measurements were correlated with FVC% and DLCO%. Densitometric measures including higher HAA%, higher MLD, lower skewness and lower kurtosis, and a higher proportion of local histogram defined interstitial changes made up of honeycombing were all associated with shorter transplant free survival. In addition, in this cohort, GAP scores that utilized quantitative CT measures of interstitial disease (GAP-QCT, GAP-honeycombing and GAP-HAA) performed similarly to the standard GAP score and to the visual analysis based GAP-CT score with regard to associations with transplant free survival.

A multitude of variables have been evaluated as markers of disease severity in IPF, including clinical predictors such as symptom based scoring systems, spirometric and other physiologic measures, pathologic patterns, serum and bronchoalveolar lavage biomarkers, and both qualitative and quantitative radiographic measures [4]. Each of these has its advantages and disadvantages with regard to accuracy, reproducibility, ease of use, and applicability at the individual patient level. With regard to radiographic predictors, both qualitative scoring systems and the visual presence of a UIP pattern have been associated with a higher risk of death [6, 24, 28–33]. While useful, these approaches are limited by the need trained experts, the time consuming process of visual review, and the subjectivity of visual analysis. For instance, Walsh et al showed that inter-observer agreement for the presence of UIP on CT was only 0.40, even for experienced thoracic radiologists [34]. These limitations suggest a role for quantitative CT analysis, especially when considering the analysis of images from larger cohorts.

In prior studies of CT scans obtained as part of research protocols, higher values of MLD and lower values of skewness and kurtosis, have been associated with worse pulmonary function, with the absolute value of correlation coefficients ranging from 0.18 to 0.74 [10, 17, 35]. In addition, Best et al found that skewness and kurtosis were associated with mortality [6]. In our study, we replicated those results using non-volumetric, clinically acquired CT scans, suggesting that these methods may be applicable outside of a research setting. In fact, the levels of association with FVC and DLCO in particular were higher than previously described, perhaps due to the relative severity and homogeneity of disease in our cohort. In addition, we found that impairments in all of the densitometric measures that we evaluated were associated with shorter transplant free survival.

We also evaluated a local histogram and distance based approach for quantifying radiographic subtypes of lung tissue. We, and others, have used similar approaches in the study of emphysema, but there has been limited evaluation of such methods in IPF, and, to our knowledge, we are the first to apply this specific technique, which includes the distance from the pleural surface as part of the analysis, to IPF. [9, 18, 19, 36] We found that higher values of both the total percentage of lung occupied by interstitial features and the proportion of those features that were honeycombing, were strongly correlated with impairments in FVC% and DLCO%. We also found that there was a trend toward worse transplant free survival for those with higher total amounts of interstitial changes, a finding analogous to that of visual fibrosis scores. In addition, there was worse transplant free survival for those with a higher proportion of interstitial disease that was honeycombing, a finding which could be considered analogous to the visual presence of a UIP type radiographic pattern. For simplicity, we did not directly evaluate clinical associations with the percentage of total lung occupied by honeycombing, however we did find that the total percentage of lung occupied by interstitial changes was highly correlated with the percentage of those changes that were honeycombing (r = 0.95, *p* < 0.001) suggesting that the two may have similar clinical associations.

Prior work by Ley et al showed that a visual fibrosis score could substitute for DLCO, thus we sought to both replicate this finding and determine if quantitative measures could be used in place of this qualitative measurement [16]. We found that both the standard continuous GAP score and the visual GAP-CT score performed similarly to their previous descriptions, and that, when used in place of the visual score, the quantitative measures based on local histogram analysis (GAP-QCT and GAP-honeycombing) provided similar results with regard to transplant free survival. The GAP score based on the densitometric measure HAA% (GAP-HAA) performed nearly, but not quite, as well as the other quantitative CT measures. Together, these findings suggest that quantitative CT measures could be used in place of qualitative measures for predicting risk in patients with IPF, though this interpretation is limited by the exploratory nature of this study and the use of only the derivation cohort for these analyses. Additional studies in validation cohorts, including those with less severe disease, will be needed to validate this finding. Of note, for the GAP-QCT score we utilized a measure of all interstitial changes, including ground glass, while the visual fibrosis score only reflects reticular and honeycombing changes. The latter we chose based on its prior description in the literature [16]. With regard to the former, we felt that for simplicity it would be best to quantitatively characterize lung tissue as normal, emphysematous or interstitial, with the only subgroup analysis being the analysis of honeycombing due to its known visual association with poor outcomes [32]. In this cohort this difference in fibrosis/interstitial score definition is unlikely to have dramatically affected the results as the vast majority (84.1%) of the objective interstitial changes were either reticular or honeycombing, but additional work will be needed in larger and less diseased cohorts to determine what potential role each of the interstitial tissue subtypes play in the objective CT analysis of IPF.

The strengths of our study include the robustness of our findings despite the use of clinically acquired data including not excluding patients with suboptimal imaging. In addition, using the local histogram and distance based approach we were able to specifically evaluate the association of a particular radiographic tissue type with more severe disease. This method, while in many ways more complex than densitometry, provides a more intuitive result: the percentage of lung occupied by interstitial disease. This simplicity may make this method more clinically usable than other, less intuitive measures. The local histogram based approach may also be more accurate than densitometry in subjects who have both emphysema and IPF as low attenuation emphysematous areas may confound whole lung densitometric measures. Future studies will be needed to evaluate this possibility, as well as to compare the performance of this method against other densitometric, local histogram, and textural analysis based approaches [6–13, 16, 17, 37]. Finally, while the local histogram and distance based approach is a somewhat complex method to initially deploy, if validated, then it could be easily scaled to analyze as many CT scans as desired, without the need for time consuming visual reads by a single expert, and with the reproducibility that may be lacking if visual analysis is performed by multiple experts. This scalability would allow its use in potentially much larger research and clinical cohorts.

This study does have several weaknesses, including the small sample size, the single center retrospective design, the use of a cohort with biopsy proven and very severe disease, the inclusion of training cases in the final analysis, and the need to augment the automated lung segmentation with manual editing in the majority of the cases. Of particular note are the sample size, which limited our ability to adjust for other covariates in the mortality models, and the disease severity based both on the percentages of interstitial disease and the high rates of death and transplant, which potentially limit generalizability to those patients with less severe disease. The latter limitation is likely due in part to the fact that as a tertiary referral and transplant center, many of the patients we see have more advanced disease, as well as because of our choice to only include those patients with biopsy proven IPF. We chose to include only those with biopsy proven IPF to ensure that our findings were related to that disease in particular, however, it should be noted that during the visual review process it was noted that the vast majority of subjects did have visual CT findings highly consistent with IPF, suggesting that based on current guidelines a significant number of the biopsies may not have been performed. Finally, while the relatively high percentages of objectively defined interstitial changes and honeycombing seen in our study are to some extent likely due to the disease severity in the cohort, and while the visual fibrosis score and objective interstitial score were highly correlated, the large absolute difference between the objective and visual measures suggests that there is likely some over classification of normal tissue as diseased. This may be due to the significant radiographic noise present in the clinically acquired CT scans used for this study, and additional work is needed using both established and novel de-noising techniques to account for this issue [38].

In addition to the aforementioned need for replication in larger and less diseased cohorts, other areas of future research include investigating if longitudinal changes in CT measurements correlate with clinical and physiologic decline, and, if so, if they are affected in a measurable way by the new therapeutic agents for IPF. If this were the case, then these measurements may serve as valuable surrogate endpoints in clinical trials.

## Conclusions

This study of clinically acquired data in patients with biopsy proven IPF demonstrated that both densitometric and feature based quantitative CT measurements were associated in FVC% and DLCO%, as well as with transplant free survival. In addition, GAP scores that incorporate quantitative CT measures in place of either DLCO or visual CT measures are associated with transplant free survival. Further work is needed to replicate these associations in larger and less severely diseased cohorts and to determine if changes in CT measurements over time correlate with other markers of disease severity or with outcomes such as respiratory exacerbations and death.

## Additional file

Additional file 1: Technical Supplement. (ZIP 73 kb)

## Abbreviations

CT: Computed tomography
DLCO%: Percent predicted diffusing capacity for carbon monoxide
FVC%: Percent predicted forced vital capacity
GAP score: Gender age and physiology score utilizing diffusing capacity for carbon monoxide
GAP-CT score: Gender age and physiology score utilizing the percentage of lung occupied by visually identified fibrotic features in place of the diffusing capacity for carbon monoxide
GAP-HAA score: Gender age and physiology score utilizing the percentage of lung occupied by high attenuation area in place of the diffusing capacity for carbon monoxide
GAP-honeycombing score: Gender age and physiology score utilizing the percentage of interstitial features made up of the honeycombing subtype in place of the diffusing capacity for carbon monoxide
GAP-QCT score: Gender age and physiology score utilizing the percentage of lung occupied by objectively identified interstitial radiographic features in place of the diffusing capacity for carbon monoxide
HAA%: Percent high attenuating area
Honeycombing%: Percentage of objectively identified interstitial radiographic features made up by the honeycombing subtype
ILD: Interstitial lung disease
IPF: Idiopathic pulmonary fibrosis
LH: Local histogram
MLD: Mean lung density
Objective interstitial score interstitial%: Percentage of lung occupied by objectively identified interstitial radiographic features
PFT: Pulmonary function test
UIP: Usual interstitial pneumonia
Visual fibrosis score: fibrosis%, Percentage of lung occupied by visually identified interstitial features

## Footnotes

1 Note that paraseptal emphysema was specifically not included because of prior experience which revealed its frequent misclassification as normal tissue, and that no panlobular emphysema was identified during the training process.
