# Supplementary material for: Densitometric and local histogram based analysis of computed tomography images in patients with idiopathic pulmonary fibrosis
Source: Respir Res. 2017 Mar 7;18:45. doi: 10.1186/s12931-017-0527-8 (PMC5340000; doi:10.1186/s12931-017-0527-8)
Supplement: Additional file 1: — Technical Supplement. (ZIP 73 kb) [file 12931_2017_527_MOESM1_ESM.zip › supplemental material BWH ILD manuscript 2016.12.05.docx]

**Additional File**

Title: Densitometric and Local Histogram Based Analysis of Computed Tomography Images in Patients with Idiopathic Pulmonary Fibrosis

Authors: Ash SY^1^*, Harmouche R^2^*, Lassala Lopez Vallejo D^1^, Villalba JA^1^, Ostridge K^3^, Gunville R^4^, Come CE^1^, Onieva Onieva J^2^, Ross JC^2^, Hunninghake GM^1^, El-Chemaly SY^1^, Doyle TJ^1^, Nardelli P^2^, Sanchez-Ferrero, GV^2^, Goldberg HJ^1^, Rosas IO^1^, San Jose Estepar R^2^† and Washko GR^1^†

^1^ Division of Pulmonary and Critical Care Medicine, Department of Medicine, Brigham and Women’s Hospital, Boston, MA

^2^ Laboratory of Mathematics in Imaging, Department of Radiology, Brigham and Women’s Hospital, Boston, MA

^3^ University Hospital Southampton, Southampton, United Kingdom

^4^ Department of Biology, Creighton University, Omaha, NE

* Contributed equally to this project

† Contributed equally to this project

**Technical Supplement**

Clinically acquired, non-volumetric, inspiratory computed tomography (CT) scans were analyzed for this study, and the lung segmentation and densitometric assessments of the lung parenchyma were performed on the inspiratory scans using the Chest Imaging Platform (CIP) (http://acil.med.harvard.edu/chest-imaging-platform).^[^[^1^](#_ENREF_1)^]^

As discussed in the primary manuscript, the objective detection and quantification of the volume of interstitial lung features was performed using an approach similar to that designed for subtypes of emphysema.^[^[^2^](#_ENREF_2)^]^ This method combines information regarding the properties of local tissue with the distance from the pleural surface.

In order to build a library of points to be used as tissue classifiers, which in turn would be used to train the objective quantification tool, a single expert placed 3357 fiducials, in 30 randomly selected subjects, on radiologic features unique to each disease type, including normal parenchyma, interstitial subtypes (reticular, centrilobular nodule, linear scar, nodular, subpleural line, ground glass), and emphysema subtypes (centrilobular and panlobular). The subtype distribution of the training ROI can be found in e-Table 1. Note that paraseptal emphysema was specifically not included because of prior experience which revealed its frequent misclassification as normal tissue, and that no panlobular emphysema was identified during the training process.

Regions of interest (ROI) consisting of 30 by 30 in-plane voxels were constructed around each of these training points. Feature vectors consisting of a measurement of the distance from the ROI to the nearest point on the pleural surface and the local density histogram obtained using kernel density estimation (KDE) were built for each ROI. KDE is a non-parametric method used to estimate a probability distribution over the densitometry values in each region of interest by smoothing over the local histogram information (e-Figure 1). This is especially useful when a parametric distribution cannot be fit over the densitometry values. The estimated distribution ($\hat{f}$) as a function of densitometry value ($x$) was obtained by smoothing a normalized histogram of all patch samples ($x_{i}$) using a Gaussian kernel ($K$). The smoothing factor $(h)$ is particularly useful due to the finite number of densitometry samples per patch. Higher values of $h$ result in increased smoothing of the distribution and the optimal value for this factor was obtained using methods described previously.[[3](#_ENREF_3), [4](#_ENREF_4)]

$$\hat{f}\left( x \right)=\frac{1}{nh}+\sum_{i=1}^{\infty} K(\frac{x- x_{i}}{h})$$

After the training process was completed, de-novo regions of the CT scan were classified based on their similarity to the training data. Subjects included in the training set were not excluded from this analysis. For each test region, the local histogram and distance feature vectors were extracted and compared to the feature vectors of each region in the training data. The following metric that combines the *L_1_* norm between the local density histograms and a weighted difference between the distances to the chest wall was used for the comparison:

$$Density Metric=L_{1}\left( training histogram,test histogram \right)+ \beta\times L_{1}\left( training distance,test distance \right)$$

The weight ($\beta$) was determined by searching the parameter space, and in this data set, a weight of 0.013 provided the best results. A k-nearest neighbor classification scheme was then used to select the label with the highest frequency from the 5 nearest training neighbors as determined by the distance metric.

The performance of the tool was validated using a leave-one-out validation on the 3357 regions of interest, whereby at each iteration one of the regions was selected as the testing data and the remaining patches were selected to be part of the training data. The overall average accuracy was 47.7.% (e-Table 2). Further review of the data shows that the accuracy of the algorithm was 96.8% for honeycombing and 89.6% for reticular changes, but only 21.4% for nodular changes and 29.4% for subpleural line (e-Table 2). These differing levels of accuracy were in part due to the differing numbers of training points for each subtype in the training sets. For example, while 1145 points were used to train for the honeycombing subtype, only 14 were used for the nodular subtype. The ability of our classifier to obtain a high degree of accuracy for subytpes of particular interest such as honeycombing, especially in such a difficult dataset, demonstrates its ability to classify real clinical data.

**References**

1. Estepar RS, Ross JC, Harmouche R, Onieva J, Diaz AA, Washko GR: **Chest Imaging Platform: An Open-Source Library And Workstation For Quantitative Chest Imaging.** *Am J Respir Crit Care Med* 2015, **191:**A4975.

2. Castaldi PJ, San Jose Estepar R, Mendoza CS, Hersh CP, Laird N, Crapo JD, Lynch DA, Silverman EK, Washko GR: **Distinct quantitative computed tomography emphysema patterns are associated with physiology and function in smokers.** *Am J Respir Crit Care Med*, **188:**1083-1090.

3. Mendoza CS, Washko GR, Ross JC, Diaz AA, Lynch DA, Crapo JD, Silverman EK, Acha B, Serrano C, Estepar RS: **Emphysema Quantification in a Multi-Scanner Hrct Cohort Using Local Intensity Distributions.** *Proc IEEE Int Symp Biomed Imaging* 2012**:**474-477.

4. Botev ZI, Grotowski JF, Kroese DP: **Kernel density estimation via diffusion.** *The Annals of Statistics* 2010**:**2916-2957.

**Tables:**

**e-Table 1: Number of Training Samples per Radiographic Subtype**

| **Feature** | **Number of Training Points** |
| --- | --- |
| Airway | 6 |
| Bronchiectatic Airway | 112 |
| Centrilobular Emphysema | 31 |
| Ground Glass | 301 |
| Honeycombing | 1145 |
| Linear Scar | 8 |
| Nodular | 14 |
| Nodule | 1 |
| Non Bronchiectatic Airway | 8 |
| Normal Parenchyma | 853 |
| Reticular | 844 |
| Subpleural Line | 34 |
| **Total** | 3357 |

**e-Table 2: Confusion Matrix for Predicted Radiographic Subtypes (columns) vs. Actual Radiographic Subtypes (rows)**

|  | | **Predicted Class** | | | | | | | | | | |
| --- | --- | --- | --- | --- | --- | --- | --- | --- | --- | --- | --- | --- |
|  |  | Airway | Bronchiectatic Airway | Centrilobular Emphysema | Ground Glass | Honey combing | Linear Scar | Nodular | Non Bronchiectatic Airway | Normal Parenchyma | Reticular | Subpleural Line |
| **True Class** | Airway | 0.333 | 0 | 0 | 0 | 0.667 | 0 | 0 | 0 | 0 | 0 | 0 |
|  | Bronchiectatic Airway | 0 | 0.554 | 0 | 0.054 | 0.321 | 0 | 0 | 0 | 0.054 | 0.018 | 0 |
|  | Centrilobular Emphysema | 0 | 0 | 0.484 | 0.129 | 0 | 0 | 0 | 0 | 0.323 | 0.065 | 0 |
|  | Ground Glass | 0 | 0.007 | 0.017 | 0.525 | 0.083 | 0 | 0 | 0 | 0.126 | 0.243 | 0 |
|  | Honeycombing | 0.001 | 0.005 | 0 | 0.007 | 0.968 | 0 | 0 | 0 | 0 | 0.018 | 0.001 |
|  | Linear Scar | 0 | 0.125 | 0 | 0 | 0.125 | 0.5 | 0 | 0 | 0.125 | 0.125 | 0 |
|  | Nodular | 0 | 0.286 | 0 | 0.143 | 0.214 | 0 | 0.214 | 0 | 0 | 0.143 | 0 |
|  | Non Bronchiectatic Airway | 0 | 0 | 0 | 0 | 0.25 | 0 | 0 | 0 | 0.625 | 0.125 | 0 |
|  | Normal Parenchyma | 0 | 0.001 | 0.002 | 0.018 | 0 | 0 | 0.001 | 0 | 0.954 | 0.023 | 0 |
|  | Reticular | 0 | 0 | 0 | 0.03 | 0.052 | 0 | 0 | 0 | 0.018 | 0.896 | 0.005 |
|  | SubpleuralLine | 0 | 0 | 0 | 0 | 0.118 | 0 | 0 | 0 | 0 | 0.588 | 0.294 |
|  | | average = 0.477 | | | | | | | | | | |

**e-Figures**

**e-Figure 1: Average Kernel Density Estimates vs. CT Density for each Radiographic Subtype**
