# Supplementary figures and images for: Densitometric and local histogram based analysis of computed tomography images in patients with idiopathic pulmonary fibrosis
Source: Respir Res. 2017 Mar 7;18:45. doi: 10.1186/s12931-017-0527-8 (PMC5340000; doi:10.1186/s12931-017-0527-8)

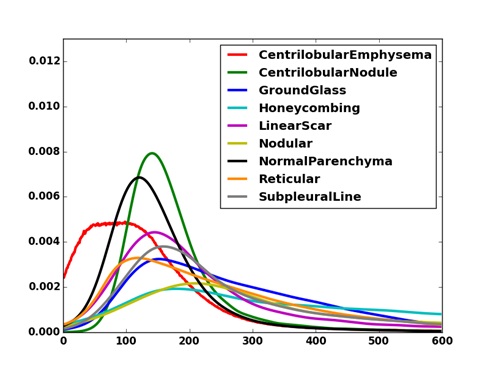

Supplement: Additional file 1: — Technical Supplement. (ZIP 73 kb) [file 12931_2017_527_MOESM1_ESM.zip › supplemental material BWH ILD figure 1 2016.06.07.jpg]
